# The Burden of Respiratory Alterations during Sleep on Comorbidities in Obstructive Sleep Apnoea (OSA)

**DOI:** 10.3390/brainsci12101359

**Published:** 2022-10-06

**Authors:** Pasquale Tondo, Francesco Fanfulla, Giulia Scioscia, Roberto Sabato, Michela Salvemini, Cosimo C. De Pace, Maria Pia Foschino Barbaro, Donato Lacedonia

**Affiliations:** 1 Department of Medical and Surgical Sciences, University of Foggia, 71122 Foggia, Italy; 2 Respiratory and Intensive Care Unit, Department of Specialistic Medicine, “Policlinico Foggia” University Hospital, 71122 Foggia, Italy; 3 Respiratory Function and Sleep Medicine Unit, Istituti Clinici Scientifici Maugeri IRCCS, 27100 Pavia, Italy

**Keywords:** apnoea–hypopnoea index, comorbidity, nocturnal hypoxemia, OSA, sleep disorders

## Abstract

Background: Obstructive sleep apnoea (OSA) has an important impact on the risk of morbidity and mortality, so we have designed the present study to understand which factor is most involved in the risk of developing a comorbidity between OSA severity and nocturnal hypoxemia. Methods: A retrospective study was conducted on 617 adult subjects who were referred to our unit for a suspicion of OSA between January 2018 and January 2020. Results: Sleep investigations performed by participants (72% male and obese in 70% of cases) reported an overall mean apnoea–hypopnoea index (AHI) of 44.0 ± 24.8 events·h^−1^. Overall, 66% were classified as severe OSA and 76% experienced nocturnal hypoxemia. By analysing the burden of OSA severity and nocturnal hypoxemia on the comorbidities risk, multivariate analysis highlighted the predominant role of age and obesity. Accordingly, after the exclusion of the older and obese participants from the analyses, we noticed that severe OSA was related to the risk of hypertension (odds ratio (OR) = 3.0 [95% confidence interval [CI] 1.4–6.2], *p* = 0.004) as well as nocturnal hypoxemia (OR = 2.6 [95% CI 1.2–5.4], *p* = 0.01). Conclusions: The study seems to suggest that in young, non-obese subjects, OSA is a predisposing factor for the risk of developing hypertension.

## 1. Introduction

Obstructive sleep apnoea (OSA) is a common sleep-related breathing disorder (SRBD) characterised by episodes of complete or partial collapse of the upper airway during the night resulting in intermittent hypoxemia [1,2,3]. These nocturnal phenomena appear to cause an increased risk of morbidity and mortality [4,5,6,7].

The diagnosis of OSA is performed through attended and unattended polysomnography (PSG) or a portable device during sleep [8,9,10,11]. The results of sleep studies show that several parameters are needed for classifying respiratory abnormalities during sleep. OSA severity is conventionally expressed in terms of the apnoea–hypopnoea index (AHI). However, another important sleep parameter used to evaluate the respiratory alterations during sleep is the sleep recording time with oxygen saturation < 90% (T_90_) that may assess the presence of gas exchange alterations, i.e., nocturnal hypoxemia (NH).

NH appears to involve a hypoxia-inducible factor (HIF), a regulator of oxygen metabolism, with activation of various pathophysiological pathways that cause the risk of developing chronic diseases such as diabetes, insulin resistance or cardiovascular diseases [12,13,14].

Overall, several studies have evaluated the correlation of SRBD with cardiovascular disease or the risk of mortality [7,15]. Other studies have assessed the link between SRBD and cancer [16,17,18,19]. Furthermore, OSA also affects the immune system [20,21], modulation of the pain response [22,23] and systemic inflammation [24].

However, although the association between OSA and the risk of developing diseases has been established by several studies, it is difficult to separate the influence of factors such as age and obesity from the risk factors closely associated with OSA itself. Moreover, as far as we know, there are no other studies that have assessed the burden of PSG parameters on the risk of chronic diseases in an Italian cohort. Therefore, we have tried to establish the burden of OSA, assessing both the severity (in terms of the high number of apnoeic or hypopnoic respiratory events during sleep) and NH, and its link with common comorbidities.

## 2. Materials and Methods

A retrospective study was conducted on adult subjects (age > 19 years) referred to our unit for SRDB-related symptoms (i.e., daytime sleepiness, snoring, etc.) between January 2018 and January 2020.

For a clinical evaluation and in the suspicion of OSA, the participants performed various medical procedures: laboratory tests, blood gas analysis, pulmonary function tests (PFTs) [25], 6-min walking test (6MWT) for evaluation of exercise performance [26]. In addition, all participants performed a cardio–respiratory monitoring (CRM). The medical history of each participant and related comorbidities were collected at clinical evaluation.

All participants after diagnosis of OSA (AHI ≥ 5 events·h^−1^ with obstructive events > 70% of global) at CRM (see sleep protocol paragraph) were included in the study. Other sleep disorders (i.e., insomnia, etc.), central sleep apnoea, subjects affected with anxiety and depression, neuromuscular diseases and overlap syndrome (OSA plus chronic obstructive pulmonary disease (COPD)) were excluded.

All procedures were conducted according to the Helsinki declaration and approved by the Ethics Committee (approval number 17/CE/2014).

### 2.1. Sleep Protocol

Each participant performed a CRM (Alice PDx, Philip Respironics, Murrysville, PA, USA) that recorded airflow with nasal cannulas and thermistors, impedance of the respiratory effort in the chest and abdomen, body position, arterial oxygen saturation (SaO_2_) and heart rate (HR).

The sleep studies were manually scored by a sleep expert physician according to the American Academy Sleep Medicine (AASM) criteria [27]. After sleep investigations, participants with OSA were classified as severe at the AHI threshold ≥ 30 events·h^−1^; indeed, NH was assessed according to the criteria of *The International Classification of Sleep Disorders*, 3rd edition (ICSD-3) [28].

### 2.2. Data Analysis

Continuous variables were expressed as mean ± standard deviation (SD) and categorical variables as percentages.

In order to find the risk factors of comorbidities, we investigated the relation of demographic variables, OSA severity and nocturnal hypoxemia with comorbidities through logistic regression analysis. In addition, the possibility of burdening the risk by developing more than three comorbidities and determining the burden using a comorbidity score (modified multisource comorbidity score (MCS)) [29] were also calculated.

To assess which sleep parameter between AHI and NH plays a decisive role as a risk factor, participants were divided into two groups: Group A (severe OSA [AHI ≥ 30 events·h^−1^] without NH assessed according to ICSD-3) and Group B (mild-to-moderate OSA [5 ≤ AHI < 30 events·h^−1^] with NH) and were compared by one-way ANalysis Of VAriance (ANOVA) or T-test, as appropriate. Then, for the two groups, we also evaluated the association with the risk of developing comorbidities.

All analyses were performed by GraphPad (GraphPad Software Inc., San Diego, CA, USA) and a *p*-value < 0.05 was considered statistically significant.

## 3. Results

In accordance with the inclusion criteria, 617 participants were enrolled in our study after the sleep study.

The cohort was mainly male (72%) and obese (body mass index (BMI) ≥ 30 kg·m^−2^ in 70% overall), with a mean age of 59.3 ± 13.5 years.

Table 1 summarises the general characteristics of the cohort.

The main comorbidities reported by the cohort were hypertension (64% overall), chronic cardiac disease (i.e., arrhythmia, valve disease, cardiomyopathy, coronary artery disease, heart failure; ~30% overall) and diabetes (23% overall).

Sleep studies performed by all participants resulted in a mean AHI of 44.0 ± 24.8 events·h^−1^, a mean ODI (oxygen desaturation index) of 41.5 ± 26.2 events·h^−1^, a mean SaO_2_ of 78.7 ± 13.1% and a mean T_90_ of 26.4 ± 29.0%. According to AHI, 66% of the population were classified as severe. In addition, 76% overall experienced NH.

**Figure 1:**
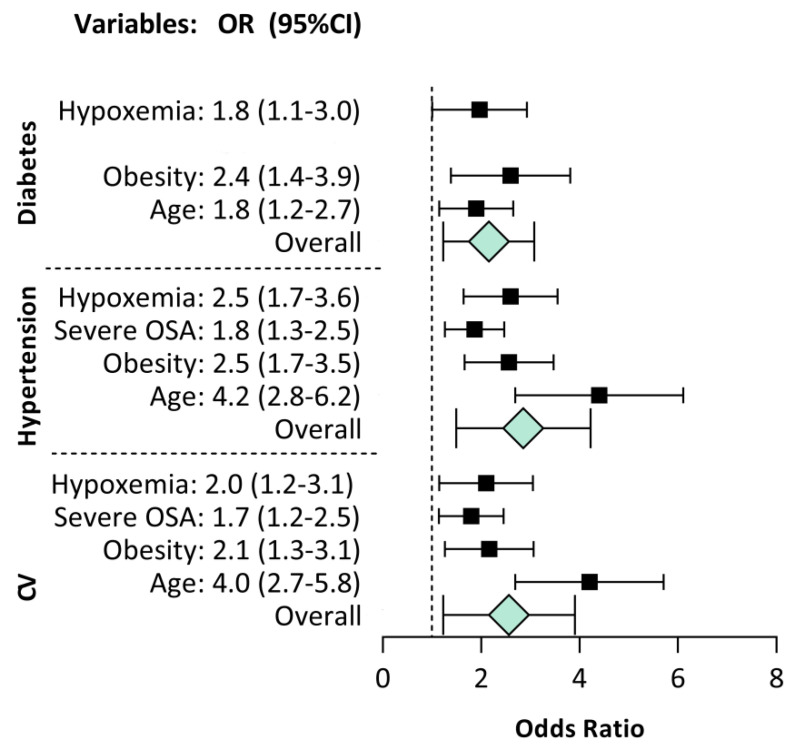
Forest plot of the odds ratio (OR) for the risk of comorbidities (cardiovascular (CV) disease, hypertension and diabetes) related to severe obstructive sleep apnoea (OSA) and nocturnal hypoxemia in the univariate analysis.

Subsequently, we have investigated the impact of the demographic and anthropometric parameters analysed (i.e., sex (male), age (≥65 years), obesity) and the sleep respiratory alterations (OSA severity and NH) on health, as shown in Figure 1.

**Figure 2:**
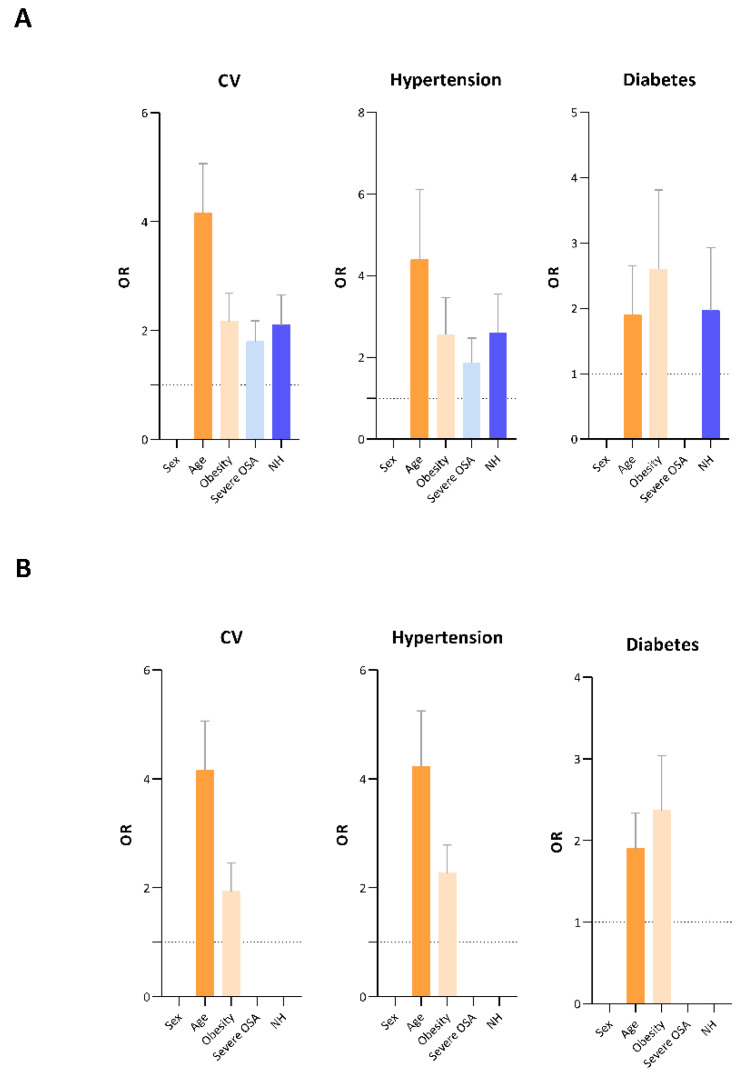
Bar plots describe the risk of developing cardiovascular diseases (CV), hypertension and diabetes by statistically significant variables analysed in (**A**) univariate logistic regression and (**B**) multivariate regression.

Univariate logistic regression analysis showed that severe OSA and NH are related to CV and hypertension; NH is also related to diabetes in contrast to severe OSA. These findings were substantially changed by multiple regression analysis. In fact, the results confirmed the burden of age and obesity on an increased risk of comorbidities even to the detriment of sleep disturbances (Table 2 and Figure 2).

Therefore, we have excluded the older (≥65 years) and obese participants and reanalysed the impact of sleep alterations on comorbidities. In this cohort, young and non-obese subjects, severe OSA (odds ratio (OR) = 3.0 [95% confidence interval [CI] 1.4–6.2], *p* = 0.004) and NH (OR = 2.6 [95% CI 1.2–5.4], *p* = 0.01) increased the risk of hypertension. Moreover, we have researched phenotypes that could play a role in the disease risk. Accordingly, the population was divided in two specific phenotypes, Group A and Group B, according to predominant sleep alteration (sleep events or NH), and then the groups were compared.

The characteristics and comparison between the two groups are summarised in Table 3.

Specifically, regarding the comorbidities suffered by the participants of two groups, no statistical differences were found. Moreover, we have not noticed a correlation between these phenotypes and a comorbidity risk.

## 4. Discussion

### 4.1. This Work and Contributions

OSA is a multifactorial disease that results in an increased incidence of the risk of developing comorbidities but also an increased risk of mortality from several factors such as cardiac causes [30]. Accordingly, we have designed the present study to analyse the association between respiratory alterations during sleep and the risk of comorbidities in an Italian cohort of subjects with OSA.

In our cohort, we found that OSA severity and NH are correlated to several comorbidities. In fact, these two factors appeared to be related to CV and hypertension.

Other studies have researched the correlation between sleep parameters and several diseases. In fact, Oldenburg et al. also investigated which nocturnal parameters between AHI, a type of SRBD (OSA or central sleep apnoea [CSA] and severity of sleep disorder) and T_90_ were associated with all-cause death. The study showed that patients with SRBD have a higher mortality than those without, risk increasing the severity index, and that T_90_ is an independent factor associated with risk and time of death [31].

Frangopoulos et al. also evaluated the association of some respiratory sleep indices with cardiovascular disease, showing that hypertension is associated with the respiratory event index (REI) >5 events·h^−1^ and >15 events·h^−^^1^, ODI > 15 events·h^−1^, mean SaO_2_ and T_90_, while arrhythmias are associated with mean SaO_2_ and T_90_ [32]. In addition, Tkacova et al. suggested that ODI is an independent predictor of hypertension rather than AHI [33].

Additionally, our study found that NH is also associated with diabetes. The mechanisms underlying this association may be different and appear to relate to repeated cycles of hypoxemia with re-oxygenation similar to an ischaemia–reperfusion injury contributing to the production of reactive oxygen species and the induction of oxidative stress. The oxidative stress increases cytokine levels with an increase in insulin resistance. Furthermore, the reduction in blood oxygen saturation stimulates the sympathetic nervous system to release catecholamines, resulting in higher serum glucose levels [34].

However, we could not ignore the main disease risk factors found in the general population, so we have adjusted our analysis for gender, old age and obesity [35,36,37].

Afterwards, no correlation was found between sleep abnormalities and comorbidities because an age of more than 65 years and obesity became the main risk factors of cardiac diseases and diabetes.

For this reason, we have investigated the possibility of developing more than three comorbidities [38] and the high risk of disease using a severity score (multisource comorbidity score) [29] in subjects with SRBD. Age and obesity were again the main risk factors.

Therefore, to understand the actual role on health of respiratory disturbances during sleep, elderly and obese subjects were excluded from our analyses. As a result, the findings showed that OSA severity and NH increase the risk of hypertension in young, non-obese population.

There are several mechanisms underlying OSA-related hypertension. Firstly, the sympathetic nervous system and the renin-angiotensin system play a key role by altering the vascular structure and consequently its function, leading to an increase in blood pressure. Intermittent hypoxia causes increased sensitivity of carotid chemoreceptors with the induction of oxidative stress and the production of angiotensin-II and endothelin-1. Angiotensin-II plays an inhibitory role on nitric oxide (NO), which in turn inhibits carotid body chemosensitivity. Accordingly, a vicious circle is triggered with hypoxia increasing the chemosensitivity of the carotid body and decreasing the bioavailability of NO. In addition, endothelin-1 acts as an excitatory agent on carotid chemoreceptors and is a vasoconstrictor peptide, which in turn contributes to modifying the vascular structure by increasing blood pressure. Other promoters of OSA-related hypertension are reactive oxygen species that interact negatively with vasodilators produced by the endothelium (e.g., NO and prostacyclin). Finally, scientific evidence suggests a genetic predisposition by finding complex interactions between angiotensin-converting enzyme (ACE) gene polymorphisms and OSA [39,40].

However, respiratory events during sleep and NH go hand in hand, so understanding which of the two factors is decisive in disease risk stratification is extremely complex. Accordingly, we have researched two independent phenotypes (Group A and Group B) that could explain the role of each sleep alteration. Nevertheless, the two phenotypes were not found to be risk factors for disease, confirming the close association between AHI and NH.

### 4.2. Limitations and Strenghts

The study presents some limitations. Firstly, it was not designed as a longitudinal study and therefore we do not know the risk over time of developing health impairments due to SRBD. Furthermore, we used the ICSD-3 classification of NH, which in our study does not appear to have a major impact on the risk of comorbidities. This problem has been compounded by other studies that have evaluated various factors that could better assess the risk of individuals with OSA such as hypoxemic burden [41]. However, a strength of our study was attempting to establish the exact burden of OSA, and the link to hypertension in patients with no other risk factors.

### 4.3. Future Work

Longitudinal studies assessing the risk of developing disease or the risk of mortality in individuals without any risk factors other than OSA are needed in order to fully understand the burden of the disease. Furthermore, it would be interesting to search for additional factors that could estimate the importance of the OSA problem or, even more interesting, to search for the biomarkers of the disease.

## 5. Conclusions

OSA is an extremely common disease that appears to be involved in the risk of developing comorbidities and worsening concomitant clinical states. Thus, we have searched for a link between OSA and major chronic diseases. The study suggests that age and obesity are the main risk factors for chronic diseases, but in a young, normal-weight population, OSA is correlated with an increased risk of developing hypertension. Certainly, the absence of longitudinal data may have biased the results, so further studies would be useful for a better assessment of disease risk, also by evaluating other OSA-related factors.

## Figures and Tables

**Table 1:** Characteristics of population.

Total (*N* = 617)			
Continuous Var		Discrete Var	
*Demographics*			
		Sex, male%	72%
Age, years	59.3 ± 13.6	≥65 years%	39%
BMI, kg·m^−2^	34.3 ± 7.5	Obesity%	70%
Neck, cm	44.8 ± 4.3		
		Smoking habit	29%
*Comorbidities*			
		CV, %	29%
		Hypertension, %	64%
		Cerebrovascular, %	11%
		Endocrinological disorder, %	13%
		Diabetes, %	23%
		Asthma, %	8%
		Co ≥ 3	19%
MCS ^‡^, points	3.4 ± 2.7		
MCS classes	1.2 ± 0.7	MCS > Class 0	34%
*Sleep data*			
AHI, events·h^−1^	44.0 ± 24.8	Severe OSA	66%
ODI, events·h^−1^	41.5 ± 26.2		
mean SaO_2_	78.7 ± 13.1		
T_90_	26.4 ± 29.0	NH	76%
ESS score, points	12.9 ± 6.1		
*Laboratory tests*			
CRP, mg/dL	4.9 ± 3.6		
Fibrinogen, mg/dL	296.7 ± 40.0		
Homocisteine, mcmol/L	41.0 ± 33.5		
*Respiratory status*			
FVC%	93.9 ± 19.2		
FEV_1_%	92.4 ± 19.9		
FEV_1_/VC	80.7 ± 6.1		
pH	7.4 ± 0.0		
PaO_2_, mmHg	78.1 ± 11.8		
PaCO_2_, mmHg	39.5 ± 4.4		
SaO_2_%	95.4 ± 2.2		
HCO_3_^−^, mmol/L	24.4 ± 2.7		
6MWT, mt	304.4 ± 79.8		

Continuous data are expressed as means ± SD, while categorical variables as shown as percentages. ^‡^ Multisource Comorbidity Score (MCS) [29] Abbreviations: 6MWT = 6-min walking test, AHI = apnoea–hypopnoea index, BMI = body mass index, Co > 3 = more than three comorbidities reported, CRP = C-reactive protein, CV = cardiovascular disease, ESS = Epworth sleepiness scale, FVC = forced vital capacity, FEV1 = forced expiratory volume in one second, HCO_3_^−^ = bicarbonate, MCS = multisource comorbidity score, NH = nocturnal hypoxemia, ODI = oxygen desaturation index, PaCO_2_ = partial pressure of carbon dioxide, PaO2 = partial pressure of oxygen, SaO_2_ = oxygen saturation, T_90_ = total sleep time spent with SaO_2_ < 90%.

**Table 2:** Evaluation of disease risk in a population with obstructive sleep apnoea (OSA).

	Univariate											
Variables	CV		Hypertension		Cerebrovascular		Endocrinological		Diabetes		Asthma	
	OR (95% CI)	*p*	OR (95% CI)	*P*	OR (95% CI)	*p*	OR (95% CI)	*p*	OR (95% CI)	*p*	OR (95% CI)	*p*
Sex	**—**	*NS*	**—**	*NS*	**—**	*NS*	0.3 (0.2–0.4)	*<0.001*	**—**	*NS*	0.2 (0.1–0.4)	*<0.001*
Old age	4.0 (2.7–5.8)	*<0.001*	4.2 (2.8–6.2)	*<0.001*	3.4 (2.0–5.8)	*<0.001*	**—**	*NS*	1.8 (1.2–2.7)	*0.002*	**—**	*NS*
Obesity	2.1 (1.3–3.1)	*0.001*	2.5 (1.7–3.5)	*0.001*	**—**	*NS*	**—**	*NS*	2.4 (1.4–3.9)	*<0.001*	**—**	*NS*
Severe OSA	1.7 (1.2–2.5)	*0.005*	1.8 (1.3–2.5)	*0.001*	**—**	*NS*	**—**	*NS*	**—**	*NS*	**—**	*NS*
NH	2.0 (1.2–3.1)	*0.003*	2.5 (1.7–3.6)	*<0.001*	**—**	*NS*	**—**	*NS*	1.8 (1.1–3.0)	*0.014*	**—**	*NS*
	Multivariate											
	**CV**		**Hypertension**		**Cerebro**		**Endocrino**		**Diabetes**		**Asthma**	
	**OR (95% CI)**	***p***	**OR (95% CI)**	***p***	**OR (95% CI)**	***p***	**OR (95% CI)**	***P***	**OR (95% CI)**	***p***	**OR (95% CI)**	***p***
Sex	**—**	**—**	**—**	**—**	**—**	**—**	**—**	**—**	**—**	**—**	**—**	**—**
Old age	4.0 (2.7–5.8)	*<0.001*	4.0 (2.6–6.1)	*<0.001*	**—**	**—**	**—**	**—**	1.8 (1.2–2.7)	*0.003*	**—**	**—**
Obesity	1.8 (1.1–2.9)	*0.01*	2.2 (1.4–3.2)	*<0.001*	**—**	**—**	**—**	**—**	2.2 (1.3–3.6)	*0.003*	**—**	**—**
Severe OSA	**—**	*NS*	**—**	*NS*	**—**	**—**	**—**	**—**	**—**	**—**	**—**	**—**
NH	—	*NS*	**—**	*NS*	**—**	**—**	**—**	**—**	**—**	*NS*	**—**	**—**

Data are expressed as odds ratio (OR) and 95% confidence interval (95% CI). A *p*-value > 0.05 was considered statistically not significant (NS). Notes: In the univariate and multivariate analyses, several demographics, anthropometric and clinical factors were included: old age (≥65 years), obesity (BMI ≥ 30 kg·m^−2^), male sex and the presence of severe OSA (AHI ≥ 30 events·h^−1^) and NH (=nocturnal hypoxemia) at sleep investigation. Abbreviations: Cerebrovascular = cerebrovascular disorders; CV = cardiovascular disease; Endocrinological = endocrinological disorders.

**Table 3:** Comparison between two phenotypes encountered: Group A (severe OSA without NH) and Group B (mild-to-moderate OSA with NH).

Variables	Group A (*N* = 49)	Group B (*N* = 109)	*p*
Sex, male%	88%	63%	0.002
Age, years	58.8 ± 13.1	62.4 ± 11.3	0.089
≥65 years%	28%	41%	0.139
BMI, kg·m^−2^	31.1 ± 5.5	34.5 ± 8.0	0.008
Obesity%	45%	72%	0.001
Neck, cm	43.3 ± 3.0	45.2 ± 4.4	0.118
Smoking habit, %	35%	19%	0.05
*Comorbidities*			
CV, %	24%	27%	0.781
Hypertension, %	57%	66%	0.285
Cerebrovascular, %	8%	10%	0.704
Endocrinological disorder, %	12%	14%	0.797
Diabetes, %	20%	24%	0.636
Asthma, %	10%	12%	0.754
Co ≥ 3	16%	19%	0.661
MCS	2.8 ± 2.6	3.3 ± 2.8	0.234
MCS classes	1.1 ± 0.7	1.3 ± 0.7	0.10
>Class 0	24%	31%	0.394
*Sleep data*			
AHI, events·h^−1^	45.9 ± 15.3	19.1 ± 6.4	-
ODI, events·h^−1^	32.8 ± 20.7	18.8 ± 7.6	<0.001
mean SaO_2_%	88.1 ± 5.6	82.1 ± 7.3	0.005
T_90_	0.4 ± 0.5	25.8 ± 30.7	-
ESS score, points	10.1 ± 5.2	11.3 ± 6.6	0.462
*Laboratory tests*			
CRP, mg/dL	4.8 ± 3.2	5.7 ± 7.0	0.39
Fibrinogen, mg/dL	297.2 ± 39.9	301.6 ± 54.5	0.617
Homocisteine, mcmol/L	55.9 ± 59.3	38.5 ± 8.2	00.003
*Respiratory status*			
FVC%	103.2 ± 11.2	91.1 ± 20.8	0.037
FEV_1_%	104.1 ± 13.5	84.1 ± 21.6	0.001
FEV_1_/VC	82.5 ± 5.7	80.2 ± 7.6	0.214
pH	7.4 ± 0.0	7.4 ± 0.0	0.631
PaO_2_, mmHg	81.6 ± 11.7	76.4 ± 11.0	0.01
PaCO_2_, mmHg	38.4 ± 4.4	39.4 ± 4.3	0.203
SaO_2_%	95.8 ± 2.2	95.2 ± 2.3	0.13
HCO_3_^−^, mmol/L	23.8 ± 2.5	24.6 ± 2.5	0.102
6MWT, mt	302.0 ± 69.8	303.6 ± 77.8	0.899

Continuous data are expressed as means ± SD, while categorical variables are shown as percentages. Abbreviation: 6MWT = 6-min walking test, AHI = apnoea–hypopnoea index, BMI = body mass index, Co > 3 = more than three comorbidities reported, CRP = C-reactive protein, CV = cardiovascular disease, ESS = Epworth sleepiness scale, FVC = forced vital capacity, FEV1 = forced expiratory volume in one second, HCO_3_^−^ = bicarbonate, MCS = multisource comorbidity score, NH = nocturnal hypoxemia, ODI = oxygen desaturation index, PaCO_2_ = partial pressure of carbon dioxide, PaO_2_ = partial pressure of oxygen, SaO_2_ = oxygen saturation, T_90_ = total sleep time spent with SaO_2_ < 90%.

## Data Availability

Not applicable.

## Abbreviations

AHI = apnoea–hypopnoea index, BMI = body mass index, Co > 3 = more than three comorbidities reported, ESS = Epworth sleepiness scale, MCS = multisource comorbidity score, NH = nocturnal hypoxemia, ODI = oxygen desaturation index, OSA = obstructive sleep apnoea, T_90_ = total sleep time spent with SaO_2_ < 90%.
